# Eugenics Evolutionally Considered

**Published:** 1914-06

**Authors:** William Francis Waugh

**Affiliations:** Post Falls, Idaho


### Department of Eugenics.

Conducted by Dr. Malone Duggan and Dr. Theo. Y. Hull.

### THE TEXAS STATE SOCIETY OF SOCIAL HYGIENE.

Headquarters: San Antonio, Texas.

DR. MALONE DUGGAN, President, San Antonio.
PROF. A. CASWELL ELLIS, 2d Vice President, Austin.
MRS. ELI HERTZBERG, 3d Vice President, San Antonio.
DR. THEO. Y. HULL, Secretary, San Antonio.
MR. W. L. EVANS, Treasurer, San Antonio.

EXECUTIVE COMMITTEE.

Dr. Malone Duggan, Chm.
Dr. Theo. Y. Hull, Sec'y.
Dr. W. F. McCaleb.
Hon. C. H. Jenkins.
Rev. A. W. S. Garden.
Hon. J. C. Willacy.
Mr. W. L. Evans.
Dr. Frank D. Boyd.
Dr. J. R. Nichols.
Dr. J. M. McCutchan.
Dr. W. A. King.
Dr. J. W. Torbett.
Dr. F. C. Walsh.
Dr. Joe Reuss.
Dr. Frank Paschal.

### Eugenics Evolutionally Considered.

DEAR DR. DANIEL: I have read with profound interest the articles in the March "Red Back," upon eugenics. I appreciate the noble work these devoted men and women are doing and sympathize with them in the effort to make this bad old world somewhat better. Dr. Crothers' scholarly address shows well how pure living and clean thinking preserve the virility of men beyond the age at which ordinary individuals retire into the innocuous desuetude of senility—for my friend Crothers has been prominent in reform work for many a year.

Dr. Crothers looks upon the social evil from the standpoint of degeneracy. Alcoholism and its attendant and consequent evils result in the development of an innumerable host of imperfects, from whom the armies of vice, crime and disease are recruited. He has spent a lifetime in fighting this monstrous evil, this demon of man's creation, with powers for evil so diabolical as to cast into insignificance the Arcadian Demon of the Northwest Wind and all his descendants. At times people are inclined to scoff at Dr. Crothers, and to look on his startling statements as chimeric exaggeration; but the more one studies the effects of alcohol, the less is he inclined to believe that its evils can be exaggerated.

Mrs. Leats takes the very advanced position in which she has a small but highly select circle of sympathizers. That her views can never command the assent of the masses can be shown by the following considerations. Her argument is based on the hypothesis, once universally held, but now relegated to the background of man's pristine purity and his progressive decline therefrom. This doctrine has had no revival since the days of Jean Jacques Rousseau to equal the originality of that brilliant sophist. He and his followers, like Chateaubriand, St. Pierre and Marmoutel, gave us a series of pictures of youthful innocence, as in Atala, Paul and Virginia and The Incas, whose exquisite beauty and delicacy went far to commend the view of humanity on which they were based.

But this view was erroneous. Man was never innocent; there never was a golden age; and the progress of the race has been upward toward ever-advancing ideals. May I suggest to Mrs. Leats that to doubt this is to destroy the omnipotence of the God she worships and to exalt Satan as a successful rival?

To comprehend these social evils we must study man along evolutional lines. This is easy. We may trace the development of the human race by observing the growth of the child; for every child passes through all the phases of this progressive development that has conducted the race from the monad cell to the altruist, the Emerson. Thus the child early delights in tree-climbing; later he is a cave-dweller and every year some boys lose their lives in the crumbling sand of Chicago, where they are unconsciously following their troglodyte ancestors. As man developed, his primitive custom of taking what he wanted became modified, as he found other men could take his possessions as easily as he took theirs. So the idea of property and respect for its possession came to him, was forced upon him by the conditions arising from the multiplication of human beings. Thus Dame Necessity forced upon man the conviction that theft, robbery, murder and other primitive ways were unwise as retaliation was provoked thereby. In like manner marriage replaced promiscuity, and monogamy has almost supplanted the once universal polygamy.

But the course of evolution has been neither continuous nor universal. We find many men even of the most advanced races stopping at the hunter and fisher stage, and the tendency to revert to it is almost universal. Others stop at the herdsman stage in which our Aryan forbears developed that universal language from which our great Indo-European tongues have descended. For the word "daughter," identical in all these rootstock, means "milker," and denotes the place occupied by the girls in the Aryan household.

So also many men, at least in youth, have not as yet developed past the robber stage. Note how the youthful desperado, as he grows older, settles down into the law-abiding citizen. Vide, Al Jennings, former train robber, now a candidate for Governor of Oklahoma.

Again, the resort to war is justly condemned by every advanced thinker; yet, witness the universal cry for arms that went up from the youth of our country at the prospect of a war a few weeks ago. Patriotism? Surely, and of the noblest sort, but—*plus* the chance for a scrap! I wonder how many of the 2100 incorrigibles mentioned by dear Mr. Teats would have been in jail had a war opened an outlet for their ebullient youth. Not that I favor war—God forbid it! I am old enough to realize that it is all Sherman asserted; but we must not expect these boys to know or feel this as we do. And here is exactly the difficulty in the way of the social reformers. They base their demands upon the loftiest ideals developed by the modern intellect, but fail to realize with how very few such ideals are possible. Chastity is not innate in man; it also is a development, like respect for truth and for property. Many a man and some women are merely unmoral, not immoral.

The remedy, as for all human ills, lies in education, in developing all the people up to the standards of one test as fast as their expanding intellects will permit. Not too fast or we will have, like Germany, to consider how to prevent the suicide of young men in dread of the entrance examinations to our universities. Every human brain will not take on an equal development, or allow of development at the same rate. As an educator for a third of a century, I see many cases where education should pause, and the active work of the producer should begin.

To me the future is full of promise. Read the novels of Tobias Smollett, and compare the manners of his day with the present. There is still far too much drinking yet, but it is not now a gentleman's duty to be carried to bed nightly dead drunk. Nor do we see drunken men and women on the city streets so frequently as we did forty years ago. The social evil is no more prevalent than it has ever been, but vice does not flaunt her plumage so openly, and the fight against it is more active, more general, more effective. God speed the army of reform. Meanwhile, there is no class, not even the priests, who have such chances to aid in this work as have the doctors. We can watch the development of the youth, and note the rise of sex-consciousness. There is a time when a wise man may do incalculable good by a warning and explaining conversation. There is a time when a judicious, non-stimulant diet, the timely use of laxatives, and even the administration of sexual sedatives, may ward off a moral debacle and tide over the patient in the time of peril.

As I write these lines for a medical journal I may be permitted to mention the vast value of solanine here, representing in the dose of one grain all the values of half an ounce of bromide, without its vital depression or its disastrous influence over the stomach or the disfiguring acne it occasions. The hemlock upon which the priests relied to insure the chastity of the neophytes in the Eleusinian mysteries is also represented in modern therapy by the hydrobromide of cicutin, a safe and effective antierotic in the hands of the physician. Also, in the beautiful yellow jessamine of the South we have an effective sedative of the amorous passion developing under the tropical sun.

I had intended to speak of the most serious obstacle to universal chastity, the growing necessity of postponing marriage. As long as men are men and women are women, the attraction of sex will be felt, and the young, alas! have neither the knowledge nor the self-control years have brought to us, to the mature men and women who can discuss these topics coolly and wisely.

William Francis Waugh, M. D.,
Post Falls, Idaho.

